# The Choice of Sports Affects Mental Rotation Performance in Adolescents

**DOI:** 10.3389/fnins.2019.00224

**Published:** 2019-03-13

**Authors:** Stefanie Pietsch, Petra Jansen, Jennifer Lehmann

**Affiliations:** Institute of Sport Science, University of Regensburg, Regensburg, Germany

**Keywords:** mental rotation, sports, object-based transformations, egocentric transformations, dancing, soccer, visual-spatial abilities

## Abstract

This study investigates mental rotation performance of adolescent female dancers and soccer players in object-based and egocentric mental rotation tasks using human body stimuli. 60 young females, 30 soccer players, and 30 dancers (not twosome), completed a chronometric mental rotation task with object-based and egocentric transformation of male and female figures, which were displayed either in front or back view. During their sport-specific activity soccer-players and dancers very often have to adapt their movements to the movement of a partner or opponent, soccer-players especially in front view positions. While for soccer-players reaction time (RT) often is crucial for sporting success, dancers mainly focus on the accuracy of their movements. Therefore, we expect significantly faster RTs for soccer players for front view stimuli but no differences between soccer players and dancers for back view stimuli. The main result was that soccer-players showed a significantly shorter RT than dancers for stimuli presented in front view in object based and egocentric transformation. There was no such difference, when the stimuli were presented in the back view. Contrary to literature we didn’t find significantly higher RTs and error rates for stimuli presented in front view compared to back view in general but only for egocentric transformations. The results of this study show that specific sports affect individual aspects of mental rotation performance.

## Introduction

Physical activity and sports not only benefit bodily constitution, but also cause positive effects on different parts of cognition (Sibley and Etnier, 2003). Enhanced physical activity promotes neuromodulation and the adaption of functional and structural properties of the human brain (Draganski et al., 2004). In sports science meta-analysis data showed a positive relation between motor and cognitive performance for children and adults (Etnier et al., 2006; Raine et al., 2018). Athletes performed better in executive functions (Vestberg et al., 2012), processing speed (Voss et al., 2009) and attention performance (Hüttermann and Memmert, 2014) compared to non-athletes. Especially in the area of visual-spatial capacity, athletes exhibited superior performance in spatial tasks compared to physically inactive people (Pietsch and Jansen, 2012; Voyer and Jansen, 2017). But most studies concerning the relation of spatial and motor performance bother with the influence of general physical activity or sports on visual-spatial abilities as a whole (Tlauka et al., 2008; Steggemann et al., 2011; Voyer and Jansen, 2017), while the influence of individual sports disciplines on different forms of mental rotation performance requires more precise research. In order to specify prior findings in this study we investigate the specific relationship between different sports and specific kinds of mental rotation tasks with embodied stimuli, namely object-based and egocentric transformations in front and back view in an adolescent sample. These results contribute to specify the relation of motor and visuo-spatial processes in greater detail and give a first hint that mental rotation performance of athletes depends on the sport- specific demands of visual perception.

Mental rotation, which is defined as the mental representation and rotation of objects (Shepard and Metzler, 1971; Linn and Petersen, 1985), is one of the best-investigated spatial abilities. Classic chronometric mental rotation tasks (cMRTs) usually contain two-dimensional (letters, animals, hands) or three-dimensional stimuli (cube figures) rotated in picture-plane and/or picture-depth, which are presented in object-based or egocentric transformation tasks (Zacks et al., 2002; Reed et al., 2006).

In object-based transformations two either identical (that is non-mirror reversed) or different (mirror-reversed) versions of a stimulus are presented side-by-side on a screen. Regarding egocentric transformations, mostly a picture of a single human figure with the left or the right arm raised and rotated in different angular disparities is presented on the middle of the screen. Typically, there exists an increase in response time with ascending angular disparity between the two presented stimuli or the presented stimulus and the stimulus in upright position (Shepard and Metzler, 1971; Wohlschläger and Wohlschläger, 1998; Jola and Mast, 2005). Stimuli presented in front view (facing the participant) resulted in higher reaction times (RTs) and error rates compared to presented in back view, facing away from the participant (Jola and Mast, 2005). This was explained by the additional in-depth rotation.

Independent of the type of stimuli (embodied vs. non-embodied) the left-right-decision participants have to reach in egocentric transformations, induces an internal, embodied experience (Steggemann et al., 2011), while same-different tasks lead to object-based transformations (Voyer et al., 2017).

Object-based and egocentric mental rotation tasks also differ concerning their processing strategies (Jola and Mast, 2005; Keehner et al., 2006). In object-based transformations participants mentally rotate the stimulus like an object with a fixed observer’s position. That means that the relationship between the environment and the participant’s egocentric frame of reference does not change. In contrast to this, in egocentric transformations participants shift their own perspective to solve the task. To make a left–right decision, participants rotate the representation of the own body (Devlin and Wilson, 2010).

Many studies proved the relation of spatial and motor skills and the trainability of spatial performance through a long term motor activity (see meta-analysis of an experts effect Voyer and Jansen, 2017): quasi-experimental studies showed that physically active people generally seem to have better visual-spatial skills than physically inactive people (Moreau et al., 2012; Pietsch and Jansen, 2012). Experimental studies further verified that there was a positive impact of manual motor training on mental rotation performance that is not bound to a trained object, but improved the process of mental rotation itself (Wiedenbauer and Jansen-Osmann, 2008). Even the practice of specific types of sport like wrestling for 10 month significantly enhanced mental rotation performance of students compared to a control group with running training (Moreau et al., 2012). However, not all types of sport seem to have the same impact on mental rotation performance. Athletes like gymnasts or wrestlers, who have to connect visuospatial and kinesthetic processes during their sporting activity exhibited a better mental rotation performance than athletes with mainly cardiovascular sporting disciplines like running (Moreau et al., 2011; Schmid et al., 2016). In contrast to this, elite team sport athletes didn’t show better mental rotation performance of human and abstract figures compared to non-athletes or recreational athletes (Jansen et al., 2012; Heppe et al., 2016).

Research has consistently demonstrated a gender gap in mental rotation performance (Geiser et al., 2008; Hambacha et al., 2014). Men outperform women in typical tasks like the Mental Rotation Test (Peters et al., 1995) which is mostly explained by biological, experiential, and individual factors (Terlecki and Newcombe, 2005; Moé and Pazzaglia, 2006; Yang et al., 2007). To investigate and control a potential influence of gender-stereotype sports on mental rotation performance, in this study we especially compared young female soccer-players and dancers. Soccer mainly sets high visual-spatial challenges on perception and anticipation in terms of the position of teammates, opponents and the ball, especially of that teammates in front view, which are potential passing partners. Further, compared to non-athletes, soccer-players showed faster RTs for mental rotation tasks with embodied stimuli (Jansen et al., 2012). In contrast to that, for dancers movement accuracy of one’s own movement compared to that of a teacher, which is positioned in back view to the dancer, is more important for learning new movement sequences, than RT.

The main goal of this study is to investigate differences in mental rotation performance of adolescence female soccer-players and dancers using object-based and egocentric human body stimuli in front and back view to specify the impact of specific forms of physical excellence on certain cognitive skills.

We expect significantly faster RTs for soccer players for front view stimuli but no differences between soccer players and dancers for back view stimuli. Therefore, we don’t generally estimate significantly shorter RTs for back view stimuli compared to front view stimuli. While soccer players have to deal with opponents in front view positions during training and competition, dancers, while learning new movement sequences, mainly view their coaches or peer dancers in back view position.

Furthermore, we hypothesize a higher accuracy rate of the dancing-group for object-based transformations tasks with stimuli presented in the back view. One of the most important tasks of adolescent dancers is to adopt new movement sequences precisely by observing and imitating the movements of a teacher who usually is positioned in the same perspective as the learning dancer.

If there is a difference in androgyny assessment between soccer players and dancers, we expect differences in mental rotation performance in favor of the soccer players especially for male stimuli. Further, we estimate significantly faster RTs for egocentric compared to object-based transformations (Kaltner et al., 2014) and an increasing RT with increasing angular disparity of the stimuli (Wohlschläger and Wohlschläger, 1998; Jola and Mast, 2005).

## Materials and Methods

### Participants

Sixty young females (30 non-twosome dancers and 30 soccer players) aged between 13 and 18 years participated in this study. Participants were recruited from different soccer and dancing sport clubs in Germany. Four participants had to be excluded from the calculation because they did also practice the sports of the other group. The “soccer group” consists of 28 girls (mean age = 15.29, *SD* = 1.35), the “dancing group” comprises 28 girls (mean age = 15.54, *SD* = 1.13). There was no difference in the mean age of both groups, *F*(1,54) = 0.558, n.s. Neither differed the groups in the amount of years, they practice their specific sports [soccer-group: *M* = 7.94, *SD* = 3.12; dancing: *M* = 9.00, *SD* = 2.73, *F*(1,54) = 1.800, n.s.], nor in the sporting hours per week [soccer-group: *M* = 4.18, *SD* = 1.47; dancing: *M* = 4.43, *SD* = 2.85), *F*(1,54) = 0.170, n.s.].

To make sure that dancers and soccer-players do not differ with respect to their cognitive abilities, the *Number Connection test* [Zahlenverbindungstest (ZVT), Oswald and Roth, 1987] was applied to measure cognitive processing speed and executive functions. The test consists of four sheets, on each sheet the numbers 1 to 90 are presented in a matrix in random order. All numbers have to be connected as accurately and fast as possible in ascending order. ZVT-scores are generated by measuring the time for connecting the numbers and are then transferred to corresponding IQ-values. The correlation with other tests of intelligence (e.g., Raven-SPM, CFT-30) moves between *r* = 0.60 to 0.80 (Vernon, 1993). The internal consistency of this test is about 0.90 to 0.95. The results showed no differences in IQ between soccer and dancing group, *F*(1,54) = 1.779, *p* = 0.188, $\eta_{p}^{2}$ = 0.032.

To measure differences in gender role identity all participants completed a German version of the Bem Sex-Role-Inventory (BSRI) (Schneider-Düker and Kohler, 1988) and a German version of the Self-Compassion-Scale (SCS) (Hupfeld and Ruffieux, 2011). The BSRI includes questions concerning self-concept and self-attribution of gender-specific features. No significant differences arose between the dancing- and soccer-group, *F*(1,54) = 3.630, *p* = 0.062, $\eta_{p}^{2}$ = 0.063. Even the SCS, a measurement tool for self-compassion which has been proven as an effective protective factor, promoting emotional resilience, showed no significant differences between the soccer- and dancing-group, *F*(1,54) = 0.921, *p* = 0.342, $\eta_{p}^{2}$ = 0.017.

None of the participants had taken part in a test of mental rotation performance before. All participants gave written informed consent prior to participation. The study was executed in accordance with the declaration of Helsinki for the guidelines of ethical considerations. Ethical approval for this study was not required in accordance with the conditions outlined by the German Research Society (DFG) where research that carries no additional risk beyond daily activities does not require Research Ethics Board Approval. We communicated all considerations necessary to assess the question of ethical legitimacy of the study.

### Material

**FIGURE 1:**
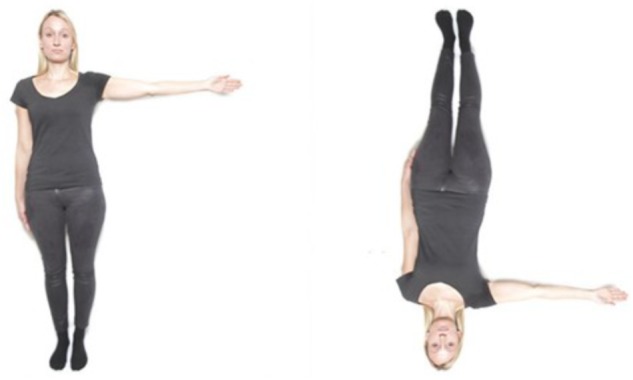
Example of the stimuli used for the different conditions. Object-based transformation of a female stimulus in front view condition (disparity of 180°). Written informed consent was obtained for the publication of this image (Kaltner and Jansen, 2018).

**FIGURE 2:**
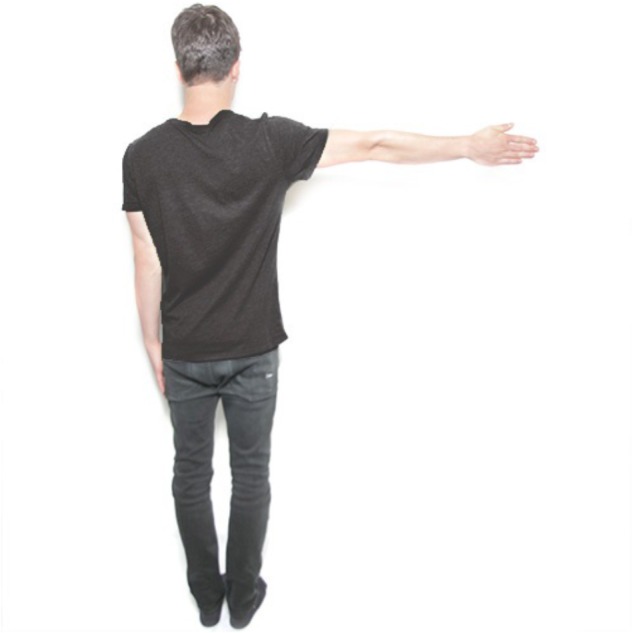
Example of the stimuli used for the different conditions. Egocentric transformation of a male stimulus in back view condition (0° condition). Written informed consent was obtained for the publication of this image.

The cMRT was conducted on a laptop with a 17^′′^ monitor, which was located approximately 60 cm in front of the participants. Stimuli were presented by using the software “Presentation” (Neurobehavioral Systems). There was an object-based and an egocentric stimulus condition with male and female stimuli presented in front and back view. In the object-based condition, two same-gender persons with either the left or the right arm raised both in front or back view were shown (see Figure 1). In the egocentric condition, one picture of a male or female person in front or back view was displayed, also with the left or the right arm raised (see Figure 2). There were two blocks of object-based and egocentric condition, with all blocks containing front and back view stimuli as well as male and female stimuli. The order of the blocks was counterbalanced.

#### Object-Based vs. Egocentric Transformations

Two male or two female figures of the same person were presented side-by-side in the center of the computer screen in the object-based condition (see Figure 1). Requiring a same-different judgment, the comparison figure on the left side was always displayed upright in the normal chirality while the right stimulus was presented in eight different angular disparities (0, 45, 90, 135, 180, 225, 270, 315°). The right figure was rotated in the picture plane in clockwise direction. Half of the trials consisted of identical pairs of objects and in the other half mirror-reversed images were presented. The left and the right stimulus always were displayed both either in front or back view. For the egocentric condition, only one male or female figure was shown in one of the eight orientations mentioned above. For half of the trials the figure raised the left arm while for the other half of the trials the figure was presented with a raised right arm (see Figure 2). Thus, a left–right decision was required from the participant.

### Procedure and Experimental Design

The individual test session, which lasted about 75 min, took place in a laboratory at University or in a quiet room in the sports club. First of all, the participants filled out a demographic questionnaire, followed by the Number Connection Test (Oswald and Roth, 1987), the FKB, the SCS, the BSRI and the cMRT (see Figure 1, 2) with a standardized task instruction. The experiment contained 12 blocks of 32 experimental trials each, resulting in 384 trials (192 object-based, 192 egocentric) in total. The order of stimulus presentation was randomized.

For the object-based conditions participants had to press the left mouse button (left click) for identical (that is only rotated) stimuli and the right mouse button (right-click) when the two stimuli were “different,” that means a mirror version of each other. For the egocentric transformations, participants pressed the left mouse button when the left arm of the figure was raised and respectively the right mouse button when the right arm was raised (see Kaltner et al., 2014).

A fixation cross was displayed at the beginning of each trial. After that, a single figure in the egocentric condition or two human stimuli in the object-based condition appeared and stayed on the screen until participants pressed the mouse button. Within the practice trials for correct responses a “+” appeared in the center of the screen and for incorrect responses a “-” appeared. The next trial began after 1500 ms. During each block, after every 32 trials a pause of 15 s was given.

### Statistical Analysis

Reaction time data were trimmed within subjects and means were calculated. Data of no participant had to be excluded because of RTs, which were longer than 2 standard deviations above the mean of the specific stimulus. As mentioned in the participants section, data of two dancers and two football-players were excluded, because in contrast to all other participants, they additionally did other sports similar to dancing (soccer-players) or soccer (dancers) besides their main sports.

Only correct trials were included in the analyses. Due to fact that the dancers and soccer-players showed no significant difference in the Bem Sex-Role-Inventory and the Self-Compassion Scale, the gender of the stimuli was not considered as a factor. Furthermore, we excluded all responses for the mirrored trials from the analysis, since for mirror-reversed responses angular disparity is not clearly defined (Jolicæur et al., 1985).

#### Influence of the Factors Angular Disparity, View and Kind of Transformation on Reaction Time and Accuracy

Two repeated measurement ANOVAs with “reaction time” and “accuracy rate” as dependent variables were conducted with the between subject factor “group” and the three within-subject factors “angular disparity” (0, 45, 90, 135, 180, 225, 270, 315°), “view” (front vs. back), and “kind of transformation” (object-based vs. egocentric). The significant interactions were analyzed further with *t*-tests. Due to the multiple-testing, we alpha-corrected in line with Bonferroni, resulting in a corrected significance level of *p* < 0.00625 for the interaction with angular disparity.

## Results

### Reaction Time

Regarding RT, repeated-measures analysis of variance showed main effects of “kind of transformation,” *F*(1,54) = 7.080, *p* = 0.01, $\eta_{p}^{2}$ = 0.116, and “angular disparity,” *F*(7,378) = 9.607, *p* < 0.005, $\eta_{p}^{2}$ = 0.151.

Furthermore, there were two two-way interactions between (a) “view” and “group,” *F*(1,54) = 4.302, *p* < 0.05, $\eta_{p}^{2}$ = 0.074, and (b) ”kind of transformation” and “view,” *F*(1,54) = 4.306, *p* = 0.05, $\eta_{p}^{2}$ = 0.074.

**FIGURE 3:**
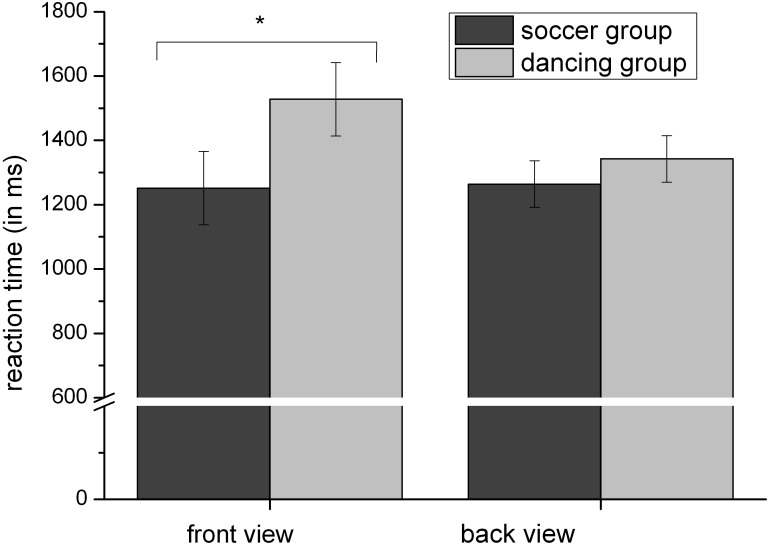
Reaction time (Mean and SE) dependent on group and view: soccer-players show significantly faster reaction times (RTs) for front view stimuli compared to dancers. ^∗^Indicates a significant difference between both groups.

Concerning our hypotheses, the two-way interaction between “view” and “group” was most relevant. The results revealed that for stimuli presented in the front view, there was a significant difference between dancers and soccer-players, *t*(54) = -2.113, *p* < 0.05. Soccer-players (*M* = 1251.49 ms, *SD* = 347.78) showed a shorter RT than dancers (*M* = 1527.99 ms, *SD* = 425.16). There was no such difference, when the stimuli were presented in the back view, *t*(54) = -0.754, *p* = 0.454, see Figure 3.

### Accuracy

We found a significant main effect for the factor “angular disparity,” *F*(7,378) = 2.348, *p* < 0.005, $\eta_{p}^{2}$ = 0.127, and a significant main effect for the factor “view,” *F*(1,54) = 12.091, *p* = 0.001, $\eta_{p}^{2}$ = 0.183. Further, there were two two-way interactions between (a) “kind of transformation” and “view,” *F*(1,54) = 71.363, *p* < 0.001, $\eta_{p}^{2}$ = 0.569 and (b) “angular disparity” and “group,” *F*(7,378) = 2.348, *p* < 0.05, $\eta_{p}^{2}$ = 0.42. Regarding the second interaction, the *t*-tests (Bonferroni corrected) showed, that only the group difference for 225° was significant, *t*(54) = -2.889, *p* = 0.006, all others failed to reach significance (all *p* > 0.625).

**FIGURE 4:**
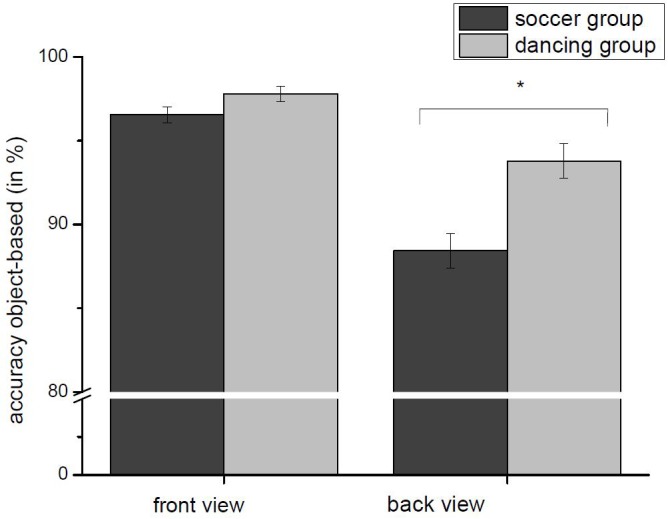
Accuracy (Mean and SE) dependent on group and view for the object-based transformation. Dancers show a significantly higher accuracy rate for back view stimuli compared to soccer-players for object-based transformation. ^∗^Indicates a significant difference between both groups.

**FIGURE 5:**
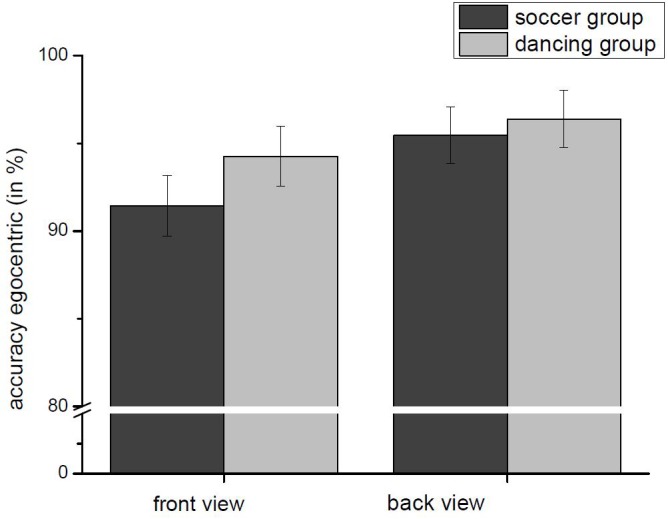
Accuracy (Mean and SE) dependent on group and view for the egocentric transformation. No significant differences between soccer-players and dancers for front and back view stimuli for egocentric transformation.

Further we found one three-way interaction between “kind of transformation,” “view,” and “group,” *F*(1,54) = 7.737, *p* < 0.005, $\eta_{p}^{2}$ = 0.125. Dancers showed a significantly higher accuracy rate for object-based stimuli presented in back view, *t*(54) = -3.654, *p* = 0.001, but not in front view, *t*(54) = -1.870, n.s.) There was no significant difference between both groups for egocentric transformations, see Figure 4 in front view, *t*(54) = -1.165, n.s., and back view, *t*(54) = -0.405, n.s., see Figure 5.

## Discussion

In the context of specific types of sports we compared mental rotation performance of female adolescents in front and back view with object-based and egocentric transformations. In contrast to previous studies (Ozel et al., 2004; Heppe et al., 2016) we matched two sports with different challenges in visual cognition concerning the position of group members and/or opponents and one’s own body posture. Additionally we tested only female soccer-players and dancers to avoid the factor of gender differences in mental rotation performance.

Regarding RT, we confirmed the frequently verified faster RTs for egocentric compared to object-based transformations and an increase of RT with increasing angular disparity (Jola and Mast, 2005). Contrary to Kaltner et al. (2014) we didn’t find a general front-view disadvantage, that means, participants didn’t generally show longer RTs for stimuli facing the participant. This holds true for object-based as well as for egocentric conditions. This finding was specified in the main and novel result of our study: soccer-players showed no difference in RT between front and back view stimuli, while dancers revealed slower RTs in front view compared to back view stimuli. Furthermore, soccer-players showed a faster RT than dancers for front view stimuli. This result suggests that the frequency and velocity in which athletes during their sporting activity have to deal with group members and/or opponents in a specific view (front/back) influences the performance in specific mental rotation tasks. While soccer players mainly have to observe facing teammates or opponents and their constantly changing distance (Jendrusch, 2009). Dancers mostly follow the movements of an instructor or group members with the same stance. For object-based as well as for egocentric transformations we found no RT difference between soccer-players and dancers. This may be due to the fact, that athletes generally show shorter RTs concerning signal transmission via visual pathways especially for complex tasks (Zwierko et al., 2010; Heirani et al., 2012). Additionally during their sporting activity both athletes have to modify their body motions to ensure an upright position during and after fast and difficult movements and have to compare their position with that of partners respectively opponents.

Furthermore, we verified a significantly higher accuracy rate of the dancing-group for object-based transformations tasks with stimuli presented in the back view. Even this result is in accord with the specific requirements of dancing. One of the most important tasks of adolescent dancers is the learning of different basic movement sequences like positions of the feet, arm postures, turns, and jumps (Schack, 2010). To precisely adopt new single and complex movement sequences it is necessary to exactly observe and imitate the movements of the dancer in front of you who usually is positioned in the same perspective as the learning dancer. Thereby the observation accuracy of different body parts is much more important than RT while imitating the new movement. Motor experts are able to adapt their movements even during fast and complex exercises on the basis of incoming perceptual information (Bardy and Laurent, 1998). Especially young dancers often practice and perform in a group and have to adapt their own movement as precisely as possible to the movement of their group members. In contrast to that, for soccer-players it is much more important to observe and react as fast as possible with a freely selectable effective movement (Andok et al., 2001), which could explain the superiority of dancers in accuracy for object-based transformations tasks with stimuli presented in the back view. This result could change for older dancers, especially ballet dancers, which frequently practice and perform long movement sequences all alone and therefore mainly concentrate on their own movements. This could effect a shift of performance in direction of egocentric transformations, which induce an internal, embodied experience (Steggemann et al., 2011). Additionally, frequent exercises in front of a mirror or a teacher instructing face to face in many cases, which normally occurs in advanced dancers, could lead to an accuracy advantage for dancers even in front view object-based transformations.

Those results certainly also would differ if we had tested twosome dancers, because they have to assimilate their dancing steps and body movements to that of their partners in front view position. Additionally types of sport without a partner like swimming or running should show the typical pattern of front-view disadvantage. Even different performances for object-based and egocentric transformation could be found, if athletes of sports without partners or opponents like high diving are compared with team sport athletes.

This is one of the first studies in motor and mental rotation development, which was conducted with adolescents. The high plasticity of the adolescent brain among others facilitates physical exercise to provoke particularly strong effects on cortical development (Konrad et al., 2013). Compared to adult athletes a high temporal amount in training is used for different basic exercises. It is one of the most important targets during the training of adolescent athletes to create a wide and profound sport specific technical and tactical basis and to develop, stabilize and modify basic movement patterns. Concerning to Munzert et al. (2009) real movements and motor mental imagery, that plays an important role for a successful motor result, share the same representations. While dancers learn new movement sequences by adjusting their own movement to that of a person, which is positioned in the same position as the dancers in front of them (back view), soccer training of adolescents contains many different forms of dribbling, passing and kicking tasks. It is one of the main goals to observe and anticipate the movement of teammates and opponents and the distance of the ball as well as to recognize passing structures and keep the orientation on the playing field (Paillard and Noé, 2005). Especially important for a successful passing or shot on goal is the position of team members and opponents, which are facing them (front view). Even during typical training exercises the opposite player mostly is situated in front view position (Brüggemann and Albrecht, 2003). Based on the sport-specific tasks of soccer, adolescent female soccer-players showed an improved mental rotation performance especially for front view tasks compared to adolescent dancers.

To extend the approach that specific types of sport affect different visual-spatial abilities the typology of spatial skills by Uttal et al. (2013) that emphasizes the intrinsic and extrinsic dimension of spatial skills as well as static and dynamic visual-spatial abilities is a valuable basis. For extrinsic visual-spatial abilities the importance of visual perception, like a better exploitation of the visual field in peripheral vision or a high binocular distance eye-sight, which is typical for game sports (Matos and Godinho, 2005; Jendrusch, 2006) is undeniable. In contrast to this, the quality of intrinsic visual-spatial abilities is based on somatosensory information, which is important in sports like artistic gymnastics or wrestling (Perrin et al., 2000). Pietsch (2018) provides a first attempt for the classification of different types of sports in a visual-spatial taxonomy for sports.

The appearance of differences in visual-spatial performance already in adolescents emphasizes actual neuroscientific findings concerning the reorganization of the brain in adolescence, which goes in line with a greater strengthening of structural and functional brain networks and improved cognitive skills (Konrad et al., 2013; Dumontheil, 2016). Our study supports the hypothesis, that specific sports make a different impact on singular visual-spatial abilities.

### Limitations

To summarize, our findings indicate, that specific aspects of mental rotation performance can be affected by different forms of physical activity. To range these results in a wider context further types of sports and even male participants have to be tested. Even the results of adult athletes especially of gender-stereotype sports have to be included. Silverman et al. (2007) ascertained that females’ spatial abilities are extremely vulnerable to attitudinal and experiential factors. While strenuous and aggressive, competitive team sports like soccer are typically estimated as male sports, dancing, gymnastics or figure skating are classified as typical female sports (Schmalz and Kerstetter, 2006). Because gender-based schematic processing has been shown to affect attitudes and behavior (Chalabaev et al., 2013), the choice of typical male or female sports should be considered as a factor for mental rotation performance. In our study the results of the Bem Sex-Role Inventory barely didn’t reach a significant level, which may be caused by the young age of the participant or the group size. Therefore, a reproduction of this study with female adults and a major sample would be desirable.

## Conclusion

Our results give a first hint that mental rotation performance of athletes depends on the sport- specific demands of visual perception. The typical RT disadvantage of front view stimuli can be erased by specific forms of motor training. The more precise examination of the impact of specific types of sports on different mental rotation tasks proved to be an interesting research topic that needs further investigation.

## Author Contributions

SP, PJ, and JL: study concept and design. SP and JL: acquisition of the data. SP and PJ: analysis and interpretation of the data. SP: drafting of the manuscript. PJ and JL: critical revision of the manuscript. PJ and SP: statistical analysis.

## Conflict of Interest Statement

The authors declare that the research was conducted in the absence of any commercial or financial relationships that could be construed as a potential conflict of interest.
